# Causes of death and early life determinants of survival in homozygous sickle cell disease: The Jamaican cohort study from birth

**DOI:** 10.1371/journal.pone.0192710

**Published:** 2018-03-01

**Authors:** Graham R. Serjeant, Nicki Chin, Monika R. Asnani, Beryl E. Serjeant, Karlene P. Mason, Ian R. Hambleton, Jennifer M. Knight-Madden

**Affiliations:** 1 Sickle Cell Trust (Jamaica), Kingston, Jamaica; 2 The Sickle Cell Unit, Caribbean Institute for Health Research, The University of the West Indies, Kingston, Jamaica; 3 The Chronic Disease Research Centre, Caribbean Institute for Health Research, The University of the West Indies, Cave Hill, Barbados; Emory University/Georgia Institute of Technology, UNITED STATES

## Abstract

Globally, the majority of persons born with sickle cell disease do not have access to hydroxyurea or more expensive interventions. The objectives were to estimate the survival in homozygous sickle cell disease, unbiased by symptomatic selection and to ascertain the causes of death in a pre-hydroxyurea population. The utility of early life biomarkers and genetically determined phenotypes to predict survival was assessed. A cohort study based on neonatal diagnosis was undertaken at the Sickle Cell Unit, a specialist clinic delivering care to persons with sickle cell disease in Jamaica. Screening of 100,000 deliveries detected 315 babies with homozygous sickle cell disease of whom 311 have been followed from birth for periods up to 43 years. Pneumococcal prophylaxis and teaching mothers splenic palpation were important, inexpensive interventions. Anticipatory guidance, routine care and out-patient acute care were provided. Each participant was classified as alive, dead, or defaulted (usually emigration). Causes of death were ascertained from clinical records and/or post-mortem reports. Survival was assessed using the Kaplan-Meier function. Sex-adjusted Cox semi-parametric proportional hazards and Weibull modelling were used to assess the effects on survival of biomarkers.

Survival to 40 years was 55.5% (95% CI 48.7% to 61.7%). Acute Chest Syndrome (n = 31) and septicemia (n = 14) were significant causes of death at all ages. Acute splenic sequestration (n = 12) was the most common cause of early deaths. Survival was significantly shorter in those with lower hemoglobin at 1 year, high total nucleated count at 1 year, and a history of dactylitis ever.

In these hydroxyurea naïve patients, survival into midlife was common. Causes of death were often age specific and some may be preventable. Early life biomarkers predictive of decreased survival in SS disease identify a patient group likely to benefit from close clinical supervision and potentially high risk therapies.

## Introduction

The interpretation of previous estimates of median survival in homozygous sickle cell (SS) disease, 42 years (males) to 48 years (females) in the US[1] and 53 years (males) to 59 years (females) in Jamaica,[2] is limited by a lack of knowledge of the overall number of patients from which these survivors are drawn. Only 11% of patients in the Jamaican study and 17% in the US study were enrolled to a clinical care facility by the age of six months. The Jamaican Cohort Study of Sickle Cell Disease, based on the follow-up of all cases detected during the screening of 100,000 consecutive non-operative deliveries, has afforded an opportunity to assess survival in a representative sample of patients. Newborn screening for sickle cell disease (SCD) commenced in June 1973. Of 315 subjects born with SS disease, 311 were recruited to the study; four could not be located.[3] The oldest are now 43 years of age and the Cohort has allowed assessment of causes of death (COD) and survival to middle age in a hydroxyurea naïve population in which, for the past 30 years, interventions have included pneumococcal prophylaxis, training caregivers in splenic palpation and provision of comprehensive health care. The predictive effects of early life biomarkers of a severe clinical course (dactylitis before age 1 year or ever, a high total nucleated cell count or low hemoglobin at age one year),[4] as well as genetically determined phenotypes (alpha-thalassemia status,[5] beta-globin haplotype[6] and fetal haemoglobin[7] were assessed. Cause/s of death was assigned, where possible

## Material and methods

Follow-up protocols for children born with SS disease required monthly assessments to 6 months, alternate months from 6 to12 months, and 3-monthly thereafter; patients being requested to attend regularly even when clinically well and were encouraged to attend at any time if sick. On each visit, special attention was paid to pain or swelling of the fingers or toes, hands or feet. Patients defaulting appointments were traced. A venipuncture was scheduled at one year. Between July and September 2016, the status of all subjects was determined as alive, dead, or emigrated. Patients were designated as emigrated when they reported this themselves or when this information was provided by family members when patients missed appointments. If dead, the probable cause of death (COD) was ascertained from medical records and/or post-mortem reports. The study was reviewed and approved by the University of the West Indies Ethics Committee. The need for informed consent for this review was waived by the Ethics Committee.

### Laboratory diagnosis & Hematological indices

The diagnosis of SS disease was based on hemoglobin electrophoresis and compatible HbA_2_ and HbF values.[8] Hematological indices were determined electronically and nucleated cell count (TNC expressed as 10^9^/L, although representing predominantly white cells, did not exclude a contribution from nucleated red cells. Fetal hemoglobin (HbF) was determined by alkali denaturation.[9] Alpha-globin genotyping was performed by Southern blot analysis.[10] Beta-globin gene haplotypes were determined by PCR-RFLP analysis of seven polymorphisms.[10,11] Since some hematological indices are age related, values for hemoglobin level, fetal hemoglobin, and nucleated cells were confined to records between 0.9–1.3 years.

#### Clinical features

Dactylitis was defined as a painful swelling of one or more digits, the hands or feet in the absence of trauma or other causes. Since dactylitis may have occurred and not been recalled over periods of patient default, those defaulting for more than one year, 13/161 (8%), were excluded from analyses of dactylitis frequency. In the others, preliminary observations on first episodes of dactylitis revealed a median age of 1.6 years (range 0.3–9.4 years) and 65 (21.8%) developed dactylitis before one year of age. Acute chest syndrome was defined as an acute onset of pulmonary symptoms and/or signs with a new pulmonary infiltrate consistent with the presence of alveolar consolidation, and excluding atelectasis.[12] Acute splenic sequestration was defined as an acute enlargement of the spleen and decrease of the hemoglobin to at least 2 g/dL below the steady state.[13] None of the patients in the Cohort received hydroxyurea. Hydroxyurea use was encouraged for severe disease after 1999 but uptake was limited by the cost of therapy and the required monitoring tests.

### Statistical methods

Survival rates at selected ages with associated 95% confidence limits were calculated using the Kaplan-Meier function. The impact on survival of alpha globin gene number, beta globin haplotype and selected early life biomarkers was then assessed using time-to-event (survival) modelling, with effect sizes presented as hazard ratios and associated 95% confidence intervals. The Kaplan-Meier function and survival modelling were used to account for censored observations, mostly due to emigrating participants.Hematological biomarkers (total nucleated cell count, total hemoglobin, and fetal hemoglobin) were selected as described closest to the first birthday and dactylitis was depicted as first dactylitis event stratified by year or at any age. The predictive effects of these biomarkers were estimated using sex-adjusted Cox semi-parametric proportional hazards models and Weibull parametric survival models. The parametric models were introduced as a brief sensitivity analysis, to explore the robustness of results to a change in methodology. These Weibull models then allowed survival predictions at different levels of each early-life predictor. Dactylitis was a time-varying discrete biomarker, and was fitted as a time-varying covariate; all other predictors were fixed effects. The frequency and median age were determined for each COD. Ninety five percent confidence intervals and exact p-values are presented where appropriate. Statistical significance was accepted as p < 0.05. All analyses were performed using Stata statistical software (StataCorp. 2017. Stata Statistical Software: Release 15. College Station, TX: StataCorp LP).

## Results

### Survival

At the time of analysis (September 2016), 119 of 311 patients were known to have died at a median age of 15.7 years (range 0.3–42.3 years), 75 had emigrated at a median age of 17.5 years (range 0.5–41.5 years), and 117 survived currently aged 34.6–42.9 years, and with a median age of 38.8 years.

Of the recruited 311 subjects, 292 (93.9%) survived to 1 year, 269 (87.1%) to 5 years, 239 (82.5%) to 10 years, 186 (74.9%) to 20 years, 152 (65.5%) to 30 years and 47 (55.2%) have so far survived to 40 years. Survival to 18 years occurred in 196 subjects (76.9%, 95% CI 71.6, 81.3) with no gender difference (female n = 90/149 survivors, 76.2%, 95% 68.3, 82.4; male n = 106/162 survivors, 77.5%, 95% CI 70.1, 83.3; log rank test, p = 0.66). The percentages represent survival proportions, calculated from the Kaplan-Meier function that accounts for censored observations; they differ from percentages calculated by simple divisions. Although half the Cohort patients have now died, the upper limit of the 95% survival confidence interval cannot yet be calculated. Current data indicate a Kaplain-Meier median survival for the whole cohort of 42.3 years (95% CI 37.2, unknown), for males of 40.2 years (95% CI 34.7, unknown) and the median for females has not yet been reached.

### Cause of death

**Fig 1:**
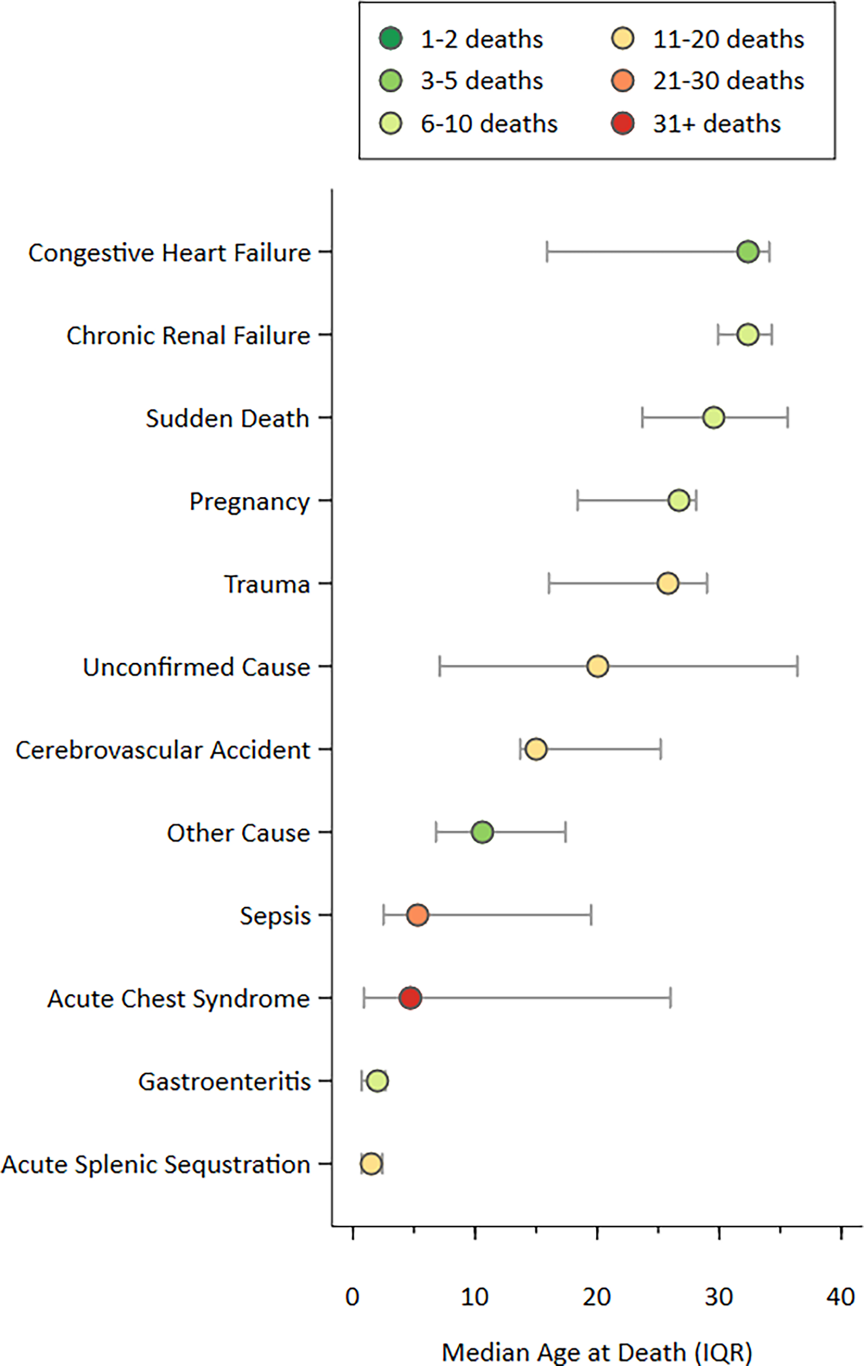
Median age at death (interquartile range) for 12 cause-of-death groups (140 causes, 119 deaths) among people with sickle cell disease.

There were 140 causes of death in 119 patients (Fig 1), recognizing multiple causes in some patients; three causes (ACS, ASS, gastroenteritis) in one patient, two causes in 20 patients, and a single causes in 98 patients. Post mortems were available for 62 (52%) of all deaths.

Acute Chest Syndrome (ACS) was the most common COD, occurring in all decades of life (n = 31; age (years, median (IQR)) = 4.7 (0.9–26.0). When ACS was one of two CODs, the second COD was sepsis (n = 4), cerebrovascular accidents (n = 2), acute splenic sequestration (ASS), congestive heart failure, pyelonephritis and HIV (n = 1 each). Pregnancy was also a factor in four deaths due to ACS. Pulmonary embolism and surgical complications each caused another death in pregnant women.

Although sepsis was a COD in all decades (n = 23), 60% (n = 14) occurred in early life (age≤6.1 years). The pathogen was identified in 18 cases; the most common pathogens were Pneumococcus (n = 7), Salmonella species (n = 5) and Hemophilus Influenzae (n = 3). Other pathogens were cultured in three cases, while sepsis was presumed clinically after partial treatment with antibiotics prior to taking cultures (n = 5). Sepsis was assumed in all cases of meningitis; one case each was associated with Pneumococcus, Salmonella and Hemophilus Influenzae and four with presumed sepsis. In one case each, sepsis was co-diagnosed with ASS and gastroenteritis (GE).

Causes of death were often age specific. ASS caused death in 10 patients in the first 3 years of life but two cases occurred at later ages (4.5 and 15.5 years respectively). Eight children died with GE before the age of four years. Eleven persons died of strokes; six in the second decade of life. Two others died of stroke in the first decade of life and three in the third.”

Trauma was the COD in twelve males; three children died in motor vehicle accidents, seven men and one woman died by violent means (ages 22–31 years). Death due to congestive heart failure and renal failure occurred primarily in the fourth decade of life.

Five patients died suddenly. Based on details related by family members, ACS and glue sniffing were suspected in one case each. Of the remaining 18 patients for whom there was no confirmed COD, the majority had some clinical data recorded during their final illness which suggested a possible COD. The COD was thought to be neurological (n = 3), due to a febrile illness (n = 2), renal, HIV related, respiratory, vascular and due to surgical complications in one case each. In eight (7%), the cause of death was completely unknown.

### Risk factors for survival

**Fig 2:**
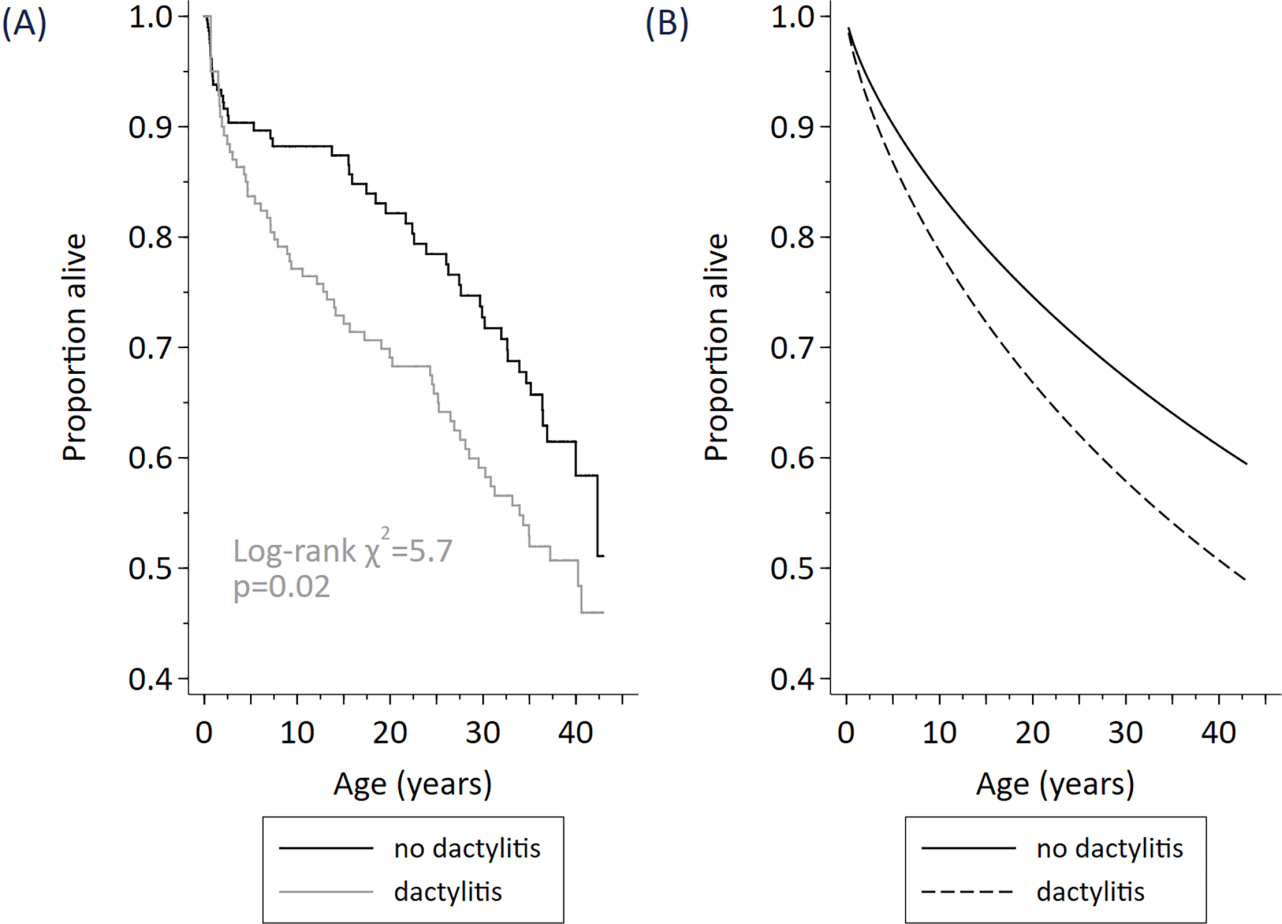
Survival among N = 298 cohort participants stratified by a history of dactylitis at any age (Fig A), modelled effect of dactylitis at any age (Fig B).

**Fig 3:**
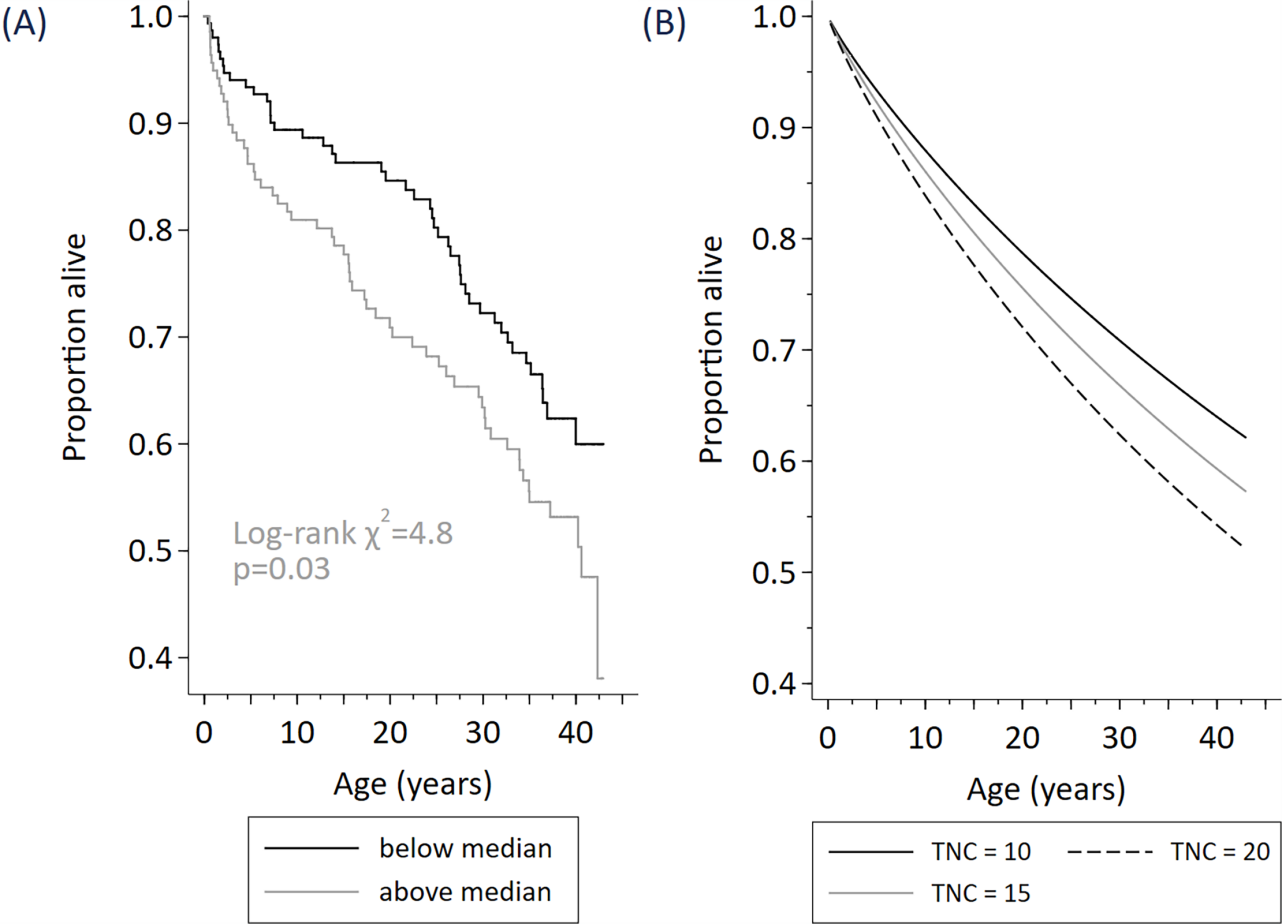
Survival among N = 290 cohort participants stratified by early life total nucleated cell count (below median / above median) (Fig A), modelled effect of total nucleated cell count (at 10x10^9^/L, 15 x10^9^/L, 20 x10^9^/L) (Fig B).

**Fig 4:**
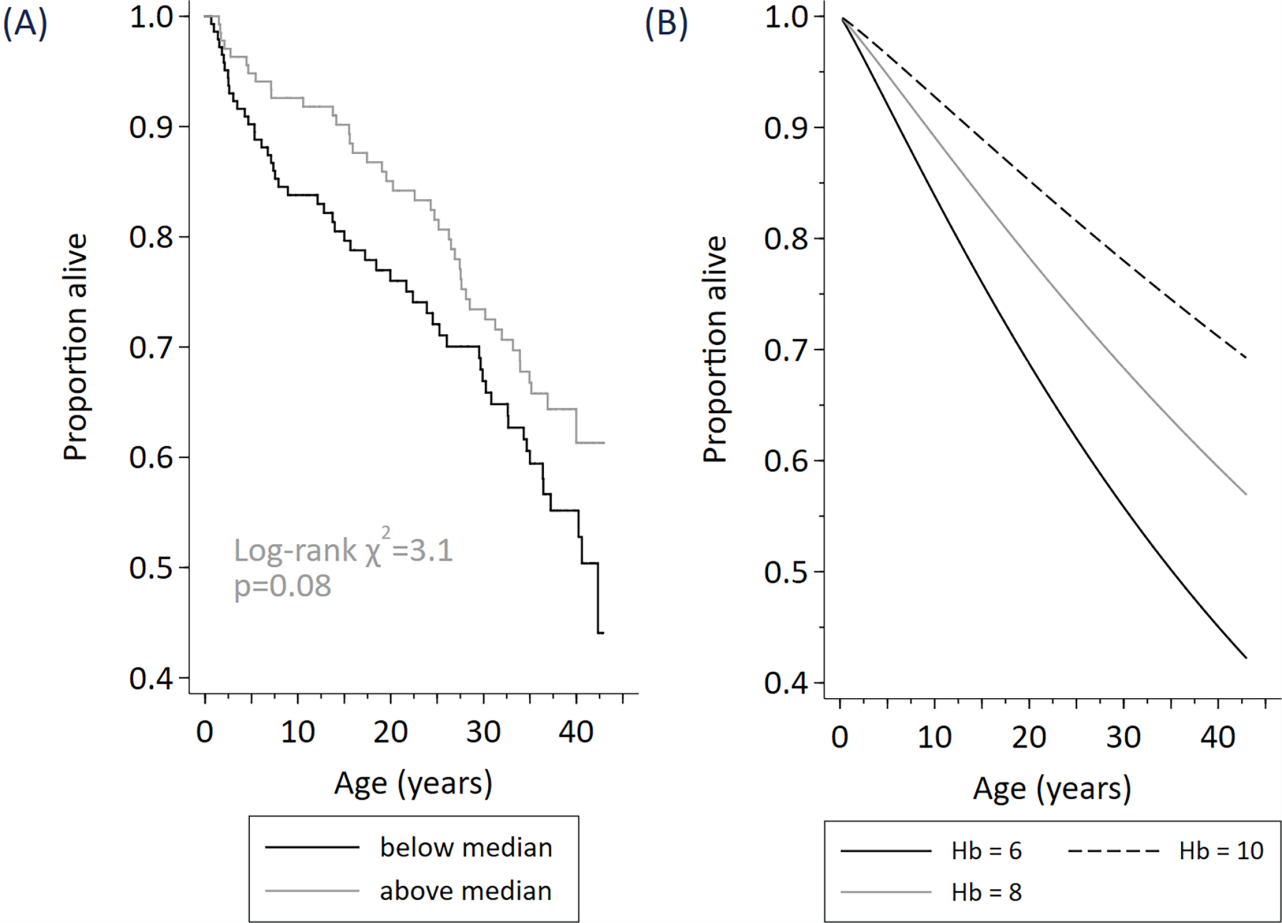
Survival among N = 279 cohort participants stratified by early life total haemoglobin level (below median / above median) (Fig A), modelled effect of total haemoglobin (at 6g/dL, 8 g/dL, 10g/dL) (Fig B).

The description of the data and hazard ratios describing the relationship of early life predictors with survival is shown (Table 1) and the results from modelling survival (Figs 2–4).

**Table 1:** Predictive effect on survival of 6 potential biomarkers, dactylitis, alpha globin gene number, beta globin haplotype, Foetal Haemoglobin (HbF), α Total Nucleated Cell Count (TNC), and Total Haemoglobin (Hb).

Predictor of survival	N	Percentage	Hazard ratio	95% CI	p-value
**Dacylitis**					
<1 year †	65	21.8	1.25	0.82, 1.92	0.31
<2 years †	119	39.9	1.22	0.84, 1.76	0.30
<3 years †	137	46.0	1.15	0.79, 1.66	0.46
<4 years †	142	47.7	1.08	0.75, 1.56	0.69
<5 years †	144	48.3	1.05	0.73, 1.52	0.78
At any age (yes/no) ‡	150	50.3	1.61	1.08, 2.39	0.02
**Alpha globin gene**					
Unknown	39				
α α / α α	172	63.2	-		0.76
α - /	91	33.5	1.03	0.65, 1.62	
α - / α -	9	3.3	1.55	0.48, 1.17	
**Beta globin haplotype**					
Unknown	92				
Benin/Benin	123	56.2	-		0.22
Benin/CAR	35	16.0	0.33	0.12, 0.94	
Benin/Senegal	17	7.8	0.87	0.31, 2.43	
Other	44	20.1	0.80	0.43, 1.50	
		**Mean (SD)**			
**HbF (%)**	246	16.3 (6.6)	0.75	0.48, 1.17	0.21
Above and below median			0.98	0.95, 1.01	0.15
Continuous (1-unit change)					
**TNC (10**^**9**^ **/ l)**	290	16.1 (7.4)			
Above and below median			1.55	1.05, 2.27	0.03
Continuous (5-unit change)			1.17	1.05, 1.31	0.01
**Hb (g/dl)**	279	8.0 (1.3)			
Above and below median			0.70	0.47, 1.05	0.11
Continuous (1-unit change)			0.81	0.69, 0.96	0.02

All estimates are sex-adjusted

† Five separate sex-adjusted Cox regressions–each dactylitis indicator included in a separate model

‡ Single sex-adjusted Cox regression with dactylitis included as a discrete time-varying variable

There was no discernible effect of alpha globin gene number, or beta globin haplotype, or fetal hemoglobin at age 1 year, but survival was shorter in those with a history of dactylitis ever and high TNC and low hemoglobin at age 1 year.

Assessed using separate gender-adjusted Cox regressions including an indicator for dactylitis by a given age (by 1-year of age, 2-years of age, and so on to 5-years of age) there was no statistically significant relationship in any model, but hazard ratios fell with increasing age. When dactylitis at any age was included in a prediction model as a time-varying indicator, dactylitis was significant at the 1% level (hazard ratio 1.61, 95% CI 1.08, 2.39, p = 0.02) (Table 1, Fig 2).

Survival was reduced among participants with higher total nucleated cell count, whether TNC was included in the prediction model as an indicator (above or below the median TNC of 14×10^9^/L), or as a continuous measure (Table 1). For example, an increase in TNC of 5×10^9^/L raised the hazard of death by 17% and so reduced survival (hazard ratio 1.17, 95% CI 1.05,1.31, p = 0.01). (Fig 3).

Survival was increased among participants with higher total hemoglobin level, whether hemoglobin was included in the prediction model as an indicator (above or below the median Hb of 8.1 g/dL), or as a continuous measure (Table 1), but only achieved statistical significance using Hb as a continuous measure. Using the prediction model, survival to 30 years of age was 56% among cohort participants with an early-life Hb of 6g/dl, was 68% with an Hb of 8g/dl, and was 78% with an Hb level of 10g/dL (Fig 4).

## Discussion

The current study reports on survival outcomes in a cohort with no exposure to hydroxyurea therapy, as did the earlier CSSCD study. Median survival in the Jamaican Cohort, although not yet confirmed, is lower than the 53 years for males and 59 years for females estimated from a Jamaican clinic based population.[2] This estimate closely matches that in the earlier Cooperative Study in the USA (Males: 42 years, Females: 48 years),[1] which was also recruited patients during in a similar period and who also had no access to hydroxyurea. This suggests that local environmental and cultural factors may not have greatly influenced mortality. Both the current estimate of survival and that from the CSSCD study were greater than a more recent American estimate based on National Center for Health Statistics multiple-cause-of-death databases[14] (Males: 33 years, Females: 37 years). The later paper found no change in mortality after the introduction of hydroxyurea and reports mortality from SCD in a variety of clinical settings, of importance, many adults did not have access to care in comprehensive centers.[14] Inevitably, there is considerable ‘noise’ in data collected over a 40 year period, partly from changes in therapy and interventions such as the introduction of pneumococcal prophylaxis,[15] and teaching mothers how to detect ASS,[16] which have changed survival,[17] but also because of causes of mortality unrelated to sickle cell disease pathology.

There were several differences in methodology between the Jamaican and Cooperative studies. The Cooperative study, based on 392 subjects (380 SS, 12 Sβ° thalassemia) ascertained before 6 months of age, may have been biased by symptomatic presentation in those not recruited by newborn screening. Patients in the Cooperative Study were derived from multiple centers with the potential weakness of observer variation whereas the Jamaican data were derived from a single center.[3,4] Dactylitis by one year of age was reported in only 41 (10%) in the Cooperative Study, compared with 65 (21.8%) in the Jamaican Cohort, probably reflecting a greater interest in recording early pathology in Jamaica. The hematology in the Cooperative Study,[4] was obtained on routine visits during the second year of life ‘when the child had no acute medical problems’ whereas the Jamaican values centered on the first birthday (range 0.9–1.3 years) and often included mild clinical conditions such as upper respiratory tract infections but excluded those associated with serious pathology or within 3 months of transfusion. Hemoglobin levels in the Cooperative Study were below 7g/dl in 22 (6%) compared with 61 (22%) in the Jamaican data: the reason for this difference being unclear. Despite these differences, lower hemoglobin levels and higher total nucleated cell counts at age 1 year were related to reduced survival in the Jamaican cohort. An elevated leucocyte count in childhood was an independent predictor of disease severity,[4] was related to the risk of stroke in the Jamaican cohort,[18] and counts above 15 x 10^9^/L were associated with an increased risk of death in patients aged 20 years or older in the Cooperative Study.[1] The mechanisms are controversial but leucocyte counts in steady state SS disease are increased and the presence of large sticky cells,[19] are likely to impair blood flow.

Dactylitis at any age was also associated with earlier death. Although dactylitis per se is not a cause of death, the underlying pathology may be indicative of vascular changes reflecting more serious pathology such as the ACS. Past studies have described the association of dactylitis with more severe outcomes in SCD.[4,20,21]

While recognizing the limited sample size from this single cohort study, potentially important negative findings were the lack of association of survival with factors previously assumed to affect expression of the disease. The effects of alpha globin gene number,[4,5,22] beta globin haplotype,[4,23] and fetal hemoglobin [24,25] on survival have reached conflicting conclusions.

These data indicate the important causes of death and their age distribution. The early causes of death in this cohort have previously been published.[17,26,27] Many COD are less common now due to improvements in care. For example, Hemophilus Influenza vaccination is now standard in all Jamaican children. Newborn screening and early childhood care were restarted at the Sickle Cell Unit in 1995 and children followed as the Jamaica Sickle Cell Unit Birth Cohort (JAMSCUB). Mortality in the first decade of life fell from 17.6% in the Jamaican Cohort to 1.8% in children in the Jamaica SCU Birth Cohort (JAMSCUB).[28] Mortality from ASS fell from 3% to 0.5% of episodes. In fact, the 5-year survival of children born with SCD from 1995 to 2006 is similar to that in the general population.[29] Audits of Pneumococcal prophylaxis reported that 88% of children with Hb SS less than four years of age were compliant with intramuscular benzathene penicillin[30] and 91% of children over four years of age had been vaccinated with the polysaccharide pneumococcal vaccine.[31] Of note, compared with the Jamaican Cohort, children in the JAMSCUB cohort were first admitted at a younger age, perhaps because parents have been trained to bring their children for attention earlier or physicians more likely to admit children who had complications earlier in the evolution of acute illnesses.

Between 1979 to 2005, mortality attributable to sickle cell disease in American children was halved to 0.5/100.000 African Americans with a decrease in deaths attributable to infections.[14] Use of national or state death records may be limited by the non-inclusion of SCD as a cause of death.[32] or as the only COD with no further details [14] Indeed, during a 2009 review of State death records, only 57.1% infants diagnosed by NBS in New York State from 2000–2008 mentioned SCD.[32] Ten of 21 deaths were thought to be SCD related and the CODs were reported as respiratory (10), sepsis (2), splenic and heart disease (one each).Many of the deaths not linked to SCD were thought to have unrelated COD, in particular prematurity and trauma. Our data also emphasizes the importance of non-SCD related CODs such as trauma.

These Jamaican data do not show the increase in mortality rates seen in the United States of America during the transition of care in early adulthood,[33] perhaps due to continuity of care at the SCU. Patients in the Jamaican Cohort Study had continuity of care when they become adults. They continued to be seen in the same facility by the same team. The patients who missed routine appointments were contacted and encouraged to return for continued care. This was in contrast to the situation in the United States of America where patients are transferred to different care teams, often in different institutions, at the time of achieving adulthood.

We do report the growing importance of chronic organ injury in older patients. Early intervention in those particularly at risk may impact chronic organ injury. For example, renal interventions in children with albuminuria, which was reported in 18% of JAMSCUB children, the youngest at 2.8 years old, may ameliorate later renal dysfunction.[34] Death associated with chronic organ disease is also more common in developed countries.[14,33] There, CODs may be different in adults with access to hydroxyurea and/or care in academic settings.[35]

The study is limited by the restricted access to some diagnostic techniques. For example, pulmonary hypertension may be unrepresented because of the unavailability of cardiac catheterization when indicated. Furthermore, the relatively high rate of emigration limits the data points contributed by some individuals.

These observations have several practical implications. Most early causes of death are preventable by careful implementation of inexpensive interventions. Furthermore, patients with risk factors for a severe clinical course or early death may benefit from closer monitoring in the clinic, and the earlier use of therapies such as hydroxyurea, one effect of which is to reduce the leucocyte count. These potential risk factors could be factored into the decision when contemplating the use of expensive and relatively high risk therapies such as bone marrow transplantation or gene therapy.
